# When BRG1 Turns Off: Chromatin Remodeling and YAP Signaling Drive ITPN Progression

**DOI:** 10.1016/j.jcmgh.2025.101676

**Published:** 2025-11-13

**Authors:** Shigetsugu Takano, Masayuki Ohtsuka

**Affiliations:** Department of General Surgery, Chiba University, Graduate School of Medicine, Chiba, Japan

Intraductal tubulopapillary neoplasm (ITPN) represents a rare and distinctive subset of pancreatic intraductal neoplasms, characterized by tubulopapillary growth with minimal mucin production. Accounting for approximately 3% of all intraductal pancreatic tumors, ITPN has increasingly gained attention as a unique precursor lesion that can progress to invasive carcinoma.^1^ Compared with conventional pancreatic ductal adenocarcinoma (PDAC), ITPN generally displays a lower malignant potential, and surgical resection often results in a favorable outcome, with a 5-year survival rate of around 70% even in patients with an invasive component.^2^ Long-term survival is achievable in most resected cases; nonetheless, the subset exhibiting invasive transformation warrants vigilant postoperative surveillance due to potential recurrence or metastasis.

The molecular pathogenesis of ITPN has emerged as an area of considerable research interest, given its distinct genetic background compared with other precursor lesions of PDAC. Although KRAS mutations are almost ubiquitous in pancreatic intraepithelial neoplasia (PanIN) and intraductal papillary mucinous neoplasms, they are notably rare in ITPN.^3^ Instead, ITPN harbors a distinct mutational landscape characterized by alterations in the PI3K/AKT signaling pathway and genes associated with chromatin remodeling. Yamaguchi et al first reported *PIK3CA* mutations in approximately 27% of ITPN cases, establishing a functional link to PI3K/AKT pathway activation.^1^ Consistent with this finding, Basturk et al subsequently identified *PIK3CA, PIK3CB, INPP4A,* and *PTEN* mutations in 27% of ITPN cases and chromatin remodeling gene alterations in 32%, underscoring the critical role of epigenetic dysregulation in ITPN oncogenesis.^4^ Furthermore, loss-of-function mutations within the switch/sucrose non-fermenting (SWI/SNF) chromatin remodeling complex, along with gain-of-function mutations in *PIK3CA*, have been recognized as frequent molecular events in this neoplasm, highlighting the interplay between chromatin architecture and oncogenic signaling in its tumorigenesis.^5^

Among the SWI/SNF complex components, the chromatin remodeling factor BRG1 (also known as SMARCA4) plays a pivotal role as a tumor suppressor in the pancreas. Von Figura and colleagues elegantly demonstrated that Brg1 loss in pancreatic ductal cells facilitates the development of intraductal papillary mucinous neoplasm and subsequent PDAC, primarily through deregulated acinar-to-ductal reprogramming and enhanced susceptibility to KRAS-driven transformation.^6^ This seminal work established BRG1 as a key gatekeeper maintaining ductal cell identity and preventing malignant conversion. However, the functional role of BRG1 loss in the context of ITPN formation has remained poorly understood.

In this context, the recent study by Iimori, Fukuda, and colleagues provides compelling new insights into the molecular mechanisms governing ITPN development.^7^ By employing sophisticated genetically engineered mouse models—notably *Hnf1b*^*CreERT2*^*; Pten*^*flox/flox*^*; Brg1*^*flox/flox*^ (HPB) mice—the authors interrogated the synergistic effects of *Brg1* and *Pten* loss within pancreatic ductal cells. Importantly, they complemented their murine findings with analyses of resected human ITPN specimens, thereby reinforcing the translational significance of their observations.

The same research group previously reported that combined loss of *Arid1a* and *Pten* in ductal cells induces ITPN formation through activation of the Yes-associated protein 1 (YAP)/transcriptional coactivator with PDZ-binding motif (TAZ) signaling pathway.^8^ Building on this foundation, the current study offers a crucial extension by demonstrating that concomitant loss of *Brg1* and *Pten* leads to an even more aggressive ITPN phenotype than that observed in *Hnf1b*^*CreERT2*^*; Pten*^*flox/flox*^*; Arid1a*^*flox/flox*^ (HPA) mice. Both HPB and HPA mice developed ITPN and ITPN-derived PDAC; however, tumor initiation and progression occurred more rapidly and extensively in the HPB cohort, implicating Brg1 loss as a key accelerator of ITPN-associated invasiveness.^7^

RNA sequencing analysis of pancreatic ductal cells from HPB mice revealed marked upregulation of YAP/TAZ signaling, suggesting that BRG1 deficiency promotes a transcriptional program conducive to tumor dedifferentiation and invasion. Notably, pharmacological inhibition of this pathway using verteporfin—a clinically available YAP inhibitor—effectively suppressed ductal dedifferentiation and tumor formation in HPB mice. This finding not only elucidates a mechanistic link between BRG1 loss and YAP/TAZ activation but also provides preclinical evidence supporting YAP-targeted therapy as a potential strategy for managing ITPN.

Collectively, this study by Iimori, Fukuda, and colleagues represents a significant advance in our understanding of ITPN biology. It integrates genetic modeling with human pathology to delineate a novel mechanistic axis—*Brg1* loss–induced activation of the YAP/TAZ pathway—driving ITPN progression. In contrast to PDAC, where BRG1 overexpression has been implicated in facilitating cancer progression, the current findings reveal that BRG1 deficiency, conversely, serves as a driver of ITPN-associated invasiveness. This dichotomy underscores the complexity of BRG1’s context-dependent roles in pancreatic tumorigenesis and highlights the necessity of refining therapeutic approaches based on molecular subtype and chromatin state.

In conclusion, this work provides a timely and thought-provoking contribution to the field of pancreatic neoplasia. By uncovering a BRG1–PI3K/AKT–YAP/TAZ regulatory axis in ITPN, the authors illuminate an uncharted dimension of chromatin–signaling crosstalk that may redefine our conceptual framework of pancreatic tumor initiation and progression. Furthermore, their demonstration of verteporfin’s antitumor efficacy against ITPN lays the groundwork for future translational research exploring YAP inhibition as a therapeutic avenue in rare but clinically significant pancreatic precursor lesions. This elegant and methodologically rigorous study not only deepens our mechanistic understanding of ITPN but also opens new prospects for precision therapy targeting the chromatin–signaling interface in pancreatic cancer biology.
